# Recurrent Stress-Induced Cardiomyopathy With Cardiogenic Shock Requiring Impella Left Ventricular Assist Device

**DOI:** 10.7759/cureus.13910

**Published:** 2021-03-15

**Authors:** Julien Feghaly, Zachary Oman, Debapria Das, Elsayed Abo-Salem

**Affiliations:** 1 Internal Medicine, Saint Louis University School of Medicine, St. Louis, USA; 2 Cardiology, Saint Louis University School of Medicine, St. Louis, USA

**Keywords:** impella, left ventricular assist device, stress induced cardiomyopathy, cardiogenic shock, impella cp, takotsubo cardioyopathy, takotsubo, device therapy in heart failure, reversible heart failure, heart failure

## Abstract

Stress-induced cardiomyopathy (SIC) is associated with varying etiologies. We present a case of a 65-year-old female with recurrent SIC secondary to seizures who presented in cardiogenic shock requiring mechanical circulatory support using an Impella CP via the right axillary approach.

## Introduction

Stress-induced cardiomyopathy (SIC) is often transient, reversible, and associated with profound physical or emotional stress [1]. There are multiple variants affecting the basal, mid, or apical left ventricular (LV) segments. The most common presentation affects the LV apex and mid-segments causing akinesia to produce the characteristic LV apical ballooning during systole, similar in shape to a Japanese ceramic octopus trap, “tako-tsubo” [2]. The clinical presentations of stress-induced cardiomyopathy are generally transient and may present as tachyarrhythmia, bradyarrhythmia, mitral regurgitation, pulmonary edema, heart failure, or cardiogenic shock requiring circulatory support [3].

In advanced cardiogenic shock refractory to drug-based therapies, mechanical circulatory support is needed. The Impella catheter is an intravascular microaxial blood pump used to provide circulatory support and can be used in both the right ventricle (RV) or the LV. When used to support the failing LV, the distal blood inlet port is positioned within the LV while the proximal blood outlet port is positioned across the aortic valve and within the aortic root. Blood is then pumped out of the LV and into the aorta unloading the ventricle and reducing ventricular wall stress. Here we present a unique case of cardiogenic shock from SIC, secondary to a neurologic etiology, supported with an Impella CP left ventricular assist device via the right axillary approach.

## Case presentation

**Figure 1:**
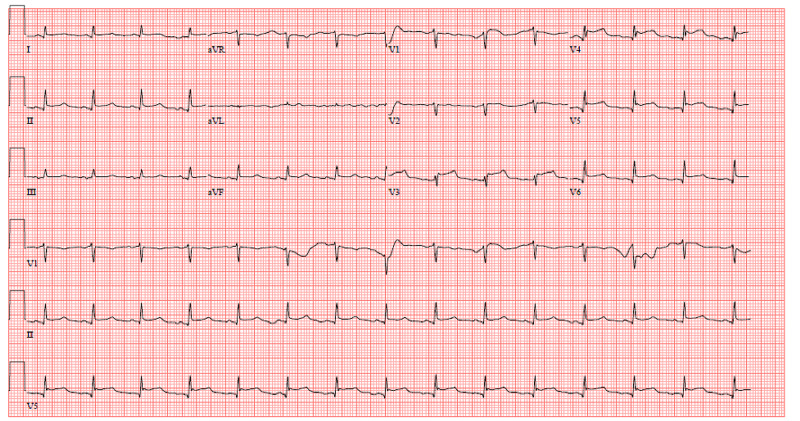
ECG demonstrating ST-elevations in inferolateral leads (V3-V6, II, aVF). ECG: electrocardiogram.

A 65-year-old female with a past medical history of hypertension, rheumatoid arthritis on antimetabolites, adrenal insufficiency secondary to chronic steroid use, and seizure disorder presented with acute onset encephalopathy associated with jerking movement of her right upper extremity. Interestingly, she had a similar presentation two-years prior with anterior and inferolateral ST-elevations on electrocardiogram (ECG) (Figure 1), normal coronary angiography, and echocardiography (ECHO) revealing SIC with ejection fraction (EF) 45%. That presentation was attributed to SIC secondary to seizure with posterior reversible encephalopathy syndrome.

**Figure 2:**
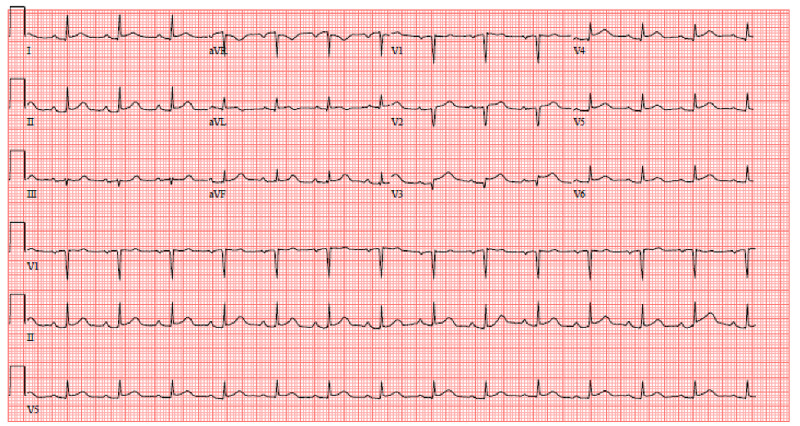
ECG demonstrating ST-elevations in V2-V5. ECG: electrocardiogram.

Upon this presentation, she was started on anti-seizure medications and empiric antibiotics for concern for meningitis. Her brain MRI was notable for multiple areas of acute infarcts in bilateral cerebral hemispheres while her ECG revealed ST-segment elevations in V2-V5 (Figure 2).

**Figure 3:**
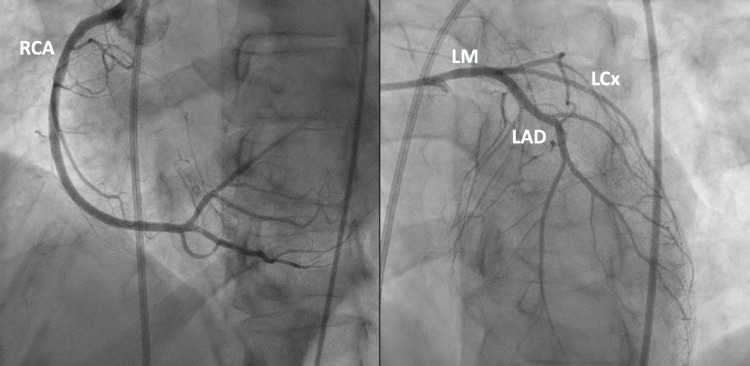
Coronary angiography. - Left main coronary artery (LM): normal size artery with minor luminal irregularities. - Left anterior descending (LAD): Large caliber artery with mild disease (<20%) gives off a first diagonal branch of a small caliber with mild disease (20%) near the ostium. The second diagonal is a medium caliber with mild disease (<20%) near the ostium. - Left circumflex (LCx): Non-dominant. A medium caliber vessel gives off a high diagonal, all with mild disease (<20%). - Right coronary artery (RCA): Large caliber vessel has a proximal lesion (<30%) then continues with minor luminal irregularities then bifurcates into the right posterior descending artery and posterolateral branches.

On presentation, she had blood pressure (BP) of 75/51 mmHg and a heart rate (HR) of 83 beats per minute (bpm). Lactic acid was 1.3 mmol/L (normal < 2.2 mmol/L). Arterial blood gas revealed metabolic acidosis on 4L oxygen via nasal cannula: pH 7.23, pCO2 38 mmHg, pO2 194 mmHg, HCO3 15.5 mmol/L. The troponin rose from 0.097 to 6.328 ng/mL (normal < 0.032 ng/mL). No kidney injury was present and the creatinine was at the patient's baseline (1.0 mg/dL). No acute liver injury was present. She was initially started on Norepinephrine (0.27 mcg/kg/min), Vasopressin (0.04 units/min), and Dobutamine (8 mcg/kg/min) to maintain a mean arterial pressure (MAP) of 65 mmHg. Coronary angiography revealed mild non-obstructive CAD (Figure 3).

**Figure 4:**
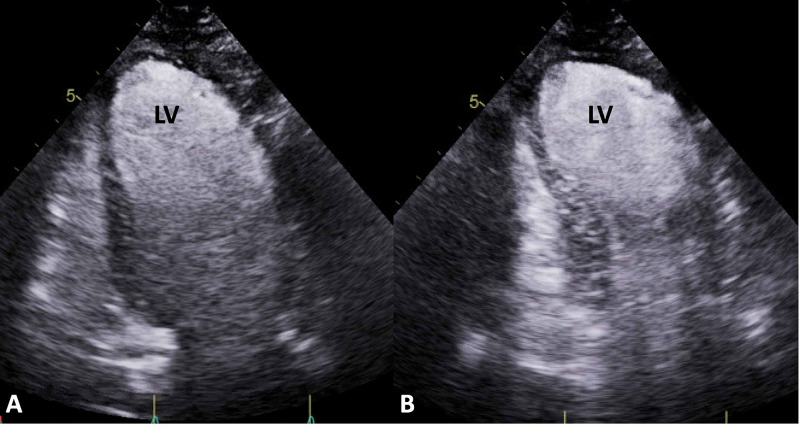
Transthoracic echocardiogram (ECHO). (A) Transthoracic ECHO with contrast in apical two-chamber view during diastole. (B) Transthoracic ECHO with contrast in apical two-chamber view during systole demonstrating left ventricle with apical ballooning and akinesis.

She was found to be in cardiogenic shock with a cardiac index of 1.2 L/min/m^2^ and cardiac output of 2.1 L/min, calculated during coronary angiography by calculating Fick’s cardiac output and index by measuring oxygen saturation in the pulmonary artery and aortic blood samples. This required the placement of a right axillary Impella CP. A right axillary approach was chosen to allow for earlier and easier mobilization and rehabilitation, compared to a femoral approach. Following Impella CP placement, Vasopressin was discontinued, and the rates of infusion were decreased for norepinephrine (0.07 mcg/kg/min) and dobutamine (6 mcg/kg/min). ECHO revealed grade 3 diastolic dysfunction, SIC, and severe global systolic dysfunction, EF 27% (Figure 4).

The following day, norepinephrine infusion was gradually decreased until discontinuation, and dobutamine infusion rate was further decreased (5 mcg/kg/min). On the second day following Impella CP placement, Dobutamine was decreased and discontinued in the morning. The flow on the Impella CP device was gradually decreased (P-8 to P-2), she remained hemodynamically stable (BP 114/68 mmHg, MAP 99 mmHg, HR 98 bpm) and the Impella CP was explanted in the afternoon on the second day. Repeat ECHO one-week later revealed LV EF of 30-35%, she remained hemodynamically stable and did not develop any subsequent end-organ injury.

She was discharged to a skilled nursing facility safely on day 13 of the admission. On follow-up after five months, she was doing well and had no readmissions. Follow-up repeat ECHO was yet to be completed. She was continued on beta-blocker and angiotensin-receptor blocker therapies for her cardiomyopathy.

## Discussion

The incidence of SIC is estimated at 7,000 to 14,000 cases in the United States, of which 90% of the cases are reported in women with mean age ranging from 58 to 75 years old [4]. These cases are thought to be associated with profound physical or emotional stress [1]. Stressors include non-cardiac surgery, myocardial dysfunction in septic proinflammatory immune states, critical illness, and neurogenic stunning in the setting of acute brain injury [4]. Recurrence of SIC has been associated with various neurologic phenomena such as status epilepticus [5], sensory and motor neuropathies [6], and transient aphasia [7]. The pathophysiology is poorly understood, but it is thought to be associated with neuroendocrine, hormonal, and vascular causes manifesting as a catecholamine surge-mediated cardiomyopathy [8].

The clinical presentations may often be difficult to distinguish from acute coronary syndrome (ACS) presenting with chest pain, dyspnea, pulmonary edema, ischemic changes on ECG or elevated troponin, and rarely presents in cardiogenic shock requiring circulatory support [4]. Only a limited number of case reports have reported the use of Impella device in patients with SIC in cardiogenic shock, with varying triggers including emotional stress [9], elective surgery [3], chemotherapy [10], or an influenza infection [11].

As with our patient, the most common abnormalities seen on ECG is ST-segment elevation in the precordial, inferior, or lateral leads, mimicking an ST-elevation myocardial infarction (STEMI) but with a lower magnitude [1, 12]. Coronary angiography tends to demonstrate normal coronary arteries or mild atherosclerosis. Apical ballooning can be seen on left ventriculography. ECHO reveals the characteristic regional wall motion abnormalities with left ventricle mid and apical hypokinesis or akinesis; in addition to the wall motion abnormalities extending beyond a single coronary artery’s distribution [4].

The diagnosis may often be challenging. Mayo Clinic proposed four criteria for diagnosing SIC: (1) transient hypokinesis, akinesis, or dyskinesis of the LV midsegments with or without apical involvement; regional wall motion abnormalities that extend beyond a single epicardial vascular distribution; a stressful trigger is frequently but not always present; (2) absence of obstructive coronary disease or angiographic evidence of acute plaque rupture; (3) new ECG abnormalities (ST-segment elevation and/or T-wave inversion) or modest elevation in cardiac troponin; and (4) absence of pheochromocytoma and myocarditis [4].

Management of SIC is mainly supportive allowing for cardiac recovery. However, patients who are in cardiogenic shock require immediate hemodynamic support to maintain adequate mean arterial pressure for end-organ perfusion. This is achieved using vasoactive medications such as catecholamines or inotropes, which pose a challenge as a catecholamine surge is a proposed etiology of SIC [8], and inotropes such as dobutamine have been associated with SIC [13]. In these situations, mechanical support devices such as the Impella can aid in providing hemodynamic support without the risks associated with vasoactive medications, reducing the myocardial workload, increasing coronary end-organ perfusion, unloading the left ventricle reducing inotropic requirements, and improving overall survival [14]. As with our patient, the Impella CP allowed for hemodynamic support as a bridge to recovery and improved EF on repeat ECHO.

## Conclusions

SIC is reversible and has been associated with various neurologic illnesses and its pathophysiology is poorly understood. SIC may manifest as ACS or cardiogenic shock requiring mechanical support. Impella CP offers hemodynamic support as a bridge to recovery with lesser complications compared to bypass MCS. Considering our patient’s outcome, it is evident that Impella left ventricular assist devices have a role to play in SIC complicated by cardiogenic shock.

## References

[1] Tsuchihashi K, Ueshima K, Uchida T (2001). Transient left ventricular apical ballooning without coronary artery stenosis: A novel heart syndrome mimicking acute myocardial infarction. J Am Coll Cardiol.

[2] Feghaly J, Mooradian A (2020). Your heart knows things your mind can’t explain. J Investig Med.

[3] Hamid T, Heart Centre M, Eichhöfer J, Fraser D, Fath-Ordoubadi F (2013). Use of the impella left ventricular assist device as a bridge to recovery in a patient with cardiogenic shock related to takotsubo cardiomyopathy. J Clin Exp Cardiol.

[4] Prasad A, Lerman A, Rihal CS (2008). Apical ballooning syndrome (Tako-Tsubo or stress cardiomyopathy): a mimic of acute myocardial infarction. Am Heart J.

[5] Legriel S, Bruneel F, Dalle L (2008). Recurrent Takotsubo cardiomyopathy triggered by convulsive status epilepticus. Neurocrit Care.

[6] Uechi Y, Higa K (2008). Recurrent Takotsubo cardiomyopathy within a short span of time in a patient with hereditary motor and sensory neuropathy. Intern Med.

[7] Sardar M, Kuntz C, Mazurek JA, Akhtar N, Saeed W, Shapiro T (2011). Recurrent takotsubo cardiomyopathy in the setting of transient neurological symptoms: a case report. J Med Case Rep.

[8] Previtali M, Repetto A, Panigada S, Camporotondo R, Tavazzi L (2009). Left ventricular apical ballooning syndrome: prevalence, clinical characteristics and pathogenetic mechanisms in a European population. Int J Cardiol.

[9] Rashed A, Won S, Saad M, Schreiber T (2015). Use of the Impella 2.5 left ventricular assist device in a patient with cardiogenic shock secondary to Takotsubo cardiomyopathy. BMJ Case Rep.

[10] Sundaravel S, Alrifai A, Kabach M, Ghumman W (2017). FOLFOX induced takotsubo cardiomyopathy treated with impella assist device. Case Reports Cardiol.

[11] Nakamura M, Nakagaito M, Hori M, Ueno H, Kinugawa K (2019). A case of Takotsubo cardiomyopathy with cardiogenic shock after influenza infection successfully recovered by IMPELLA support. J Artif Organs.

[12] Das D, Chitturu N, Feghaly J (2020). Miscoded: an imitator of a code STEMI. J Am Coll Cardiol.

[13] Mosley WJ, Manuchehry A, McEvoy C, Rigolin V (2010). Takotsubo cardiomyopathy induced by dobutamine infusion: a new phenomenon or an old disease with a new name. Echocardiography.

[14] Mariani S, Richter J, Pappalardo F (2020). Mechanical circulatory support for Takotsubo syndrome: a systematic review and meta-analysis. Int J Cardiol.

